# Supplementary material for: COVID-19 mRNA Vaccines Preserve Immunogenicity after Re-Freezing
Source: Vaccines (Basel). 2022 Apr 12;10(4):594. doi: 10.3390/vaccines10040594 (PMC9029796; doi:10.3390/vaccines10040594)
Supplement: Supplementary file 1 [file vaccines-10-00594-s001.zip › vaccines-1644328-supplementary.pdf]

Supplementary Materials

Table S1. Statistical details of experimental data are shown in Figures 2–4.

**Table S1. Effects of the administration of the Comirnaty® or Spikevax® vaccines on body weight, horizontal and vertical activities, in addition to the effects of re-freezing conditions on mRNA vaccine's integrity.**				
**Figure Number**	**Statistical Analysis**	**Factor Name**	**Statistic Value**	***p*-Value**
Figure 2B	Repeated measures ANOVA	Body weight Comirnaty®<br>Days<br>Re-freezing conditions<br>Days x Re-freezing conditions	 $F(2, 54)$= 92.242<br>$F(2, 27)$= 0.103<br>$F(4, 54)$= 0.937	 $P<0.001$<br>n.s.<br>n.s.
Figure 2C	Repeated measures ANOVA	Body weight Spikevaxy®<br>Days<br>Re-freezing conditions<br>Days x Re-freezing conditions	 $F(2, 54)$= 148.652<br>$F(2, 27)$= 0.045<br>$F(4, 54)$= 0.152	 $P<0.001$<br>n.s.<br>n.s.
Figure 2D	Repeated measures ANOVA	Horizontal activity Comirnaty® day 2<br>Days<br>Re-freezing conditions<br>Days x Re-freezing conditions<br><br>Horizontal activity Comirnaty® day 22<br>Days<br>Re-freezing conditions<br>Days x Re-freezing conditions	 $F(5, 135)$= 24.783<br>$F(2, 27)$= 1.992<br>$F(10, 135)$= 1.463<br><br><br>$F(5, 135)$= 18.983<br>$F(2, 27)$= 2.290<br>$F(10, 135)$= 2.413	 $P<0.001$<br>n.s.<br>n.s.<br><br><br>$P<0.001$<br>n.s.<br>$P<0.05$
Figure 2E	Repeated measures ANOVA	Horizontal activity Spikevaxy ® day 2<br>Days<br>Re-freezing conditions<br>Days x Re-freezing conditions<br><br>Horizontal activity Spikevaxy ® day 29<br>Days<br>Re-freezing conditions<br>Days x Re-freezing conditions	 $F(5, 135)$= 18.5671<br>$F(2, 27)$= 0.6168<br>$F(10, 135)$= 1.2094<br><br><br>$F(5, 135)$= 17.3830<br>$F(2, 27)$= 1.8675<br>$F(10, 135)$= 2.0346	 $P<0.001$<br>n.s.<br>n.s.<br><br><br>$P<0.001$<br>n.s.<br>$P<0.05$
Figure 2F	Repeated measures ANOVA	Vertical activity Comirnaty® day 2<br>Days<br>Re-freezing conditions<br>Days x Re-freezing conditions<br><br>Vertical activity Comirnaty® day 22<br>Days<br>Re-freezing conditions<br>Days x Re-freezing conditions	 $F(5, 135)$= 22.5102<br>$F(2, 27)$= 1.5546<br>$F(10, 135)$= 0.8945<br><br><br>$F(5, 135)$= 3.7228<br>$F(2, 27)$= 0.3339<br>$F(10, 135)$= 0.1493	 $P<0.001$<br>n.s.<br>n.s.<br><br><br>$P<0.01$<br>n.s.<br>n.s.
Figure 2G	Repeated measures ANOVA	Vertical activity Spikevaxy ® day 2<br>Days<br>Re-freezing conditions<br>Days x Re-freezing conditions<br><br>Vertical activity Spikevaxy ® day 29<br>Days<br>Re-freezing conditions<br>Days x Re-freezing conditions	 $F(5, 135)$= 29.6289<br>$F(2, 27)$= 1.7961<br>$F(10, 135)$= 2.3080<br><br><br>$F(5, 135)$= 4.4116<br>$F(2, 27)$= 0.0829<br>$F(10, 135)$= 0.8345	 $P<0.001$<br>n.s.<br>$P<0.05$<br><br><br>$P<0.001$<br>n.s.<br>n.s.
Figure 3A	One-way ANOVA	FU first dose Comirnaty®<br>FU second dose Comirnaty®	$F(2, 27)$= 1.756<br>$F(2, 27)$= 0.5902	n.s.<br>n.s.
Figure 3B	One-way ANOVA	% mRNA region degraded first dose Comirnaty®<br>% mRNA region degraded second dose Comirnaty®	$F(2, 27)$= 1.168<br><br>$F(2, 27)$= 2.402	n.s.<br><br>n.s.
Figure 4A	One-way ANOVA	FU first dose Spikevaxy ®<br>FU second dose Spikevaxy ®	$F(2, 27)$= 0.3449<br><br>$F(2, 27)$= 2.805	n.s.<br><br>n.s.
Figure 4B	One-way ANOVA	% mRNA region degraded first dose Spikevaxy ®<br>% mRNA region degraded second dose Spikevaxy ®	$F(2, 27)$= 1.227<br><br>$F(2, 27)$= 0.5486	n.s.<br><br>n.s.
